# Hexaaqua­magnesium bis­{4-[(5-bromo-2-hy­droxy­benzyl­idene)amino]­benzene­sulfonate} dihydrate

**DOI:** 10.1107/S1600536810053717

**Published:** 2011-01-08

**Authors:** Xi-Shi Tai

**Affiliations:** a Department of Chemistry and Chemical Engineering, Weifang University, Weifang 261061, People’s Republic of China

## Abstract

In the title hydrated mol­ecular salt, [Mg(H_2_O)_6_](C_13_H_9_BrNO_4_S)_2_·2H_2_O, the Mg^2+^ ion (site symmetry 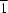) adopts a near regular MgO_6_ octa­hedral coordination geometry. In the anion, the dihedral angle between the aromatic rings is 2.5 (2)° and an intra­molecular O—H⋯N hydrogen bond generates an *S*(6) ring. In the crystal, the components are linked by O—H⋯O and O—H⋯Br hydrogen bonds.

## Related literature

For background to Schiff bases as ligands, see: Tai *et al.* (2003 ▶). 
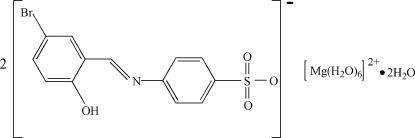


## Experimental

### 

#### Crystal data

[Mg(H_2_O)_6_](C_13_H_9_BrNO_4_S)_2_·2H_2_O
*M* *_r_* = 878.80
Monoclinic, 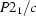

*a* = 18.7737 (14) Å
*b* = 6.2837 (5) Å
*c* = 15.7591 (12) Å
β = 108.668 (1)°
*V* = 1761.3 (2) Å^3^
*Z* = 2
Mo *K*α radiation
μ = 2.51 mm^−1^
*T* = 291 K
0.30 × 0.26 × 0.24 mm

#### Data collection

Bruker SMART APEX CCD diffractometer
Absorption correction: multi-scan (*SADABS*; Bruker, 2000 ▶) *T*_min_ = 0.48, *T*_max_ = 0.55
9144 measured reflections
3446 independent reflections
2473 reflections with *I* > 2σ(*I*)
*R*_int_ = 0.044

#### Refinement

*R*[*F* ^2^ > 2σ(*F* ^2^)] = 0.057
*wR*(*F* ^2^) = 0.131
*S* = 1.03
3446 reflections
223 parameters
H-atom parameters constrained
Δρ_max_ = 0.29 e Å^−3^
Δρ_min_ = −0.46 e Å^−3^

### 

Data collection: *SMART* (Bruker, 2000 ▶); cell refinement: *SAINT* (Bruker, 2000 ▶); data reduction: *SAINT*; program(s) used to solve structure: *SHELXTL* (Sheldrick, 2008 ▶); program(s) used to refine structure: *SHELXTL*; molecular graphics: *SHELXTL*; software used to prepare material for publication: *SHELXTL*.

## Supplementary Material

Crystal structure: contains datablocks global, I. DOI: 10.1107/S1600536810053717/hb5780sup1.cif

Structure factors: contains datablocks I. DOI: 10.1107/S1600536810053717/hb5780Isup2.hkl

Additional supplementary materials:  crystallographic information; 3D view; checkCIF report

## Figures and Tables

**Table 1:** Selected bond lengths (Å)

Mg1—O12	2.031 (3)
Mg1—O11	2.065 (3)
Mg1—O13	2.077 (3)

**Table 2:** Hydrogen-bond geometry (Å, °)

*D*—H⋯*A*	*D*—H	H⋯*A*	*D*⋯*A*	*D*—H⋯*A*
O1—H1*A*⋯N1	0.96	1.73	2.570 (5)	144
O5—H5*D*⋯O3^i^	0.85	2.00	2.854 (4)	180
O5—H5*A*⋯O4^ii^	0.85	2.08	2.892 (4)	160
O11—H11*A*⋯Br1^iii^	0.96	2.60	3.539 (3)	166
O11—H11*C*⋯O3^ii^	0.96	1.94	2.710 (4)	136
O12—H12*A*⋯O2^iv^	0.96	2.04	2.747 (4)	129
O12—H12*B*⋯O5	0.96	1.90	2.729 (4)	143
O13—H13*C*⋯O4^v^	0.96	1.88	2.763 (4)	152

Symmetry codes: (i) 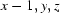; (ii) 
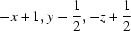
; (iii) 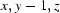; (iv) 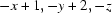; (v) 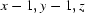.

**Fig. 1.**
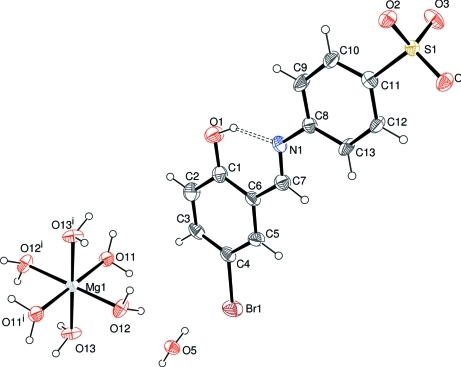
The molecular structure of (I) showing 50% displacement ellipsoids. Symmetry code: (i) –x, 1–y, –z.

## supplementary crystallographic information

### Experimental

1 mmol of magnesium perchlorate was added to a solution of 5-bromosalicylaldehyde-4-aminobenzene sulfonic acid (1 mmol) in 10 ml of 95% ethanol. The mixture was stirred for 3 h at refluxing temperature. Evaporating some ethanol, clear blocks of (I) were obtained after two weeks.

### Refinement

The H atoms were placed geometrically (C—H = 0.93–0.96 Å, O—H = 0.82 Å, N—H = 0.86 Å) and refined as riding with *U*_iso_(H) = 1.2*U*_eq_(carrier) or 1.5*U*_eq_(methyl C).

### Crystal data

**Table:**

[Mg(H_2_O)_6_](C_13_H_9_BrNO_4_S)_2_·2H_2_O	*F*(000) = 892
*M**_r_* = 878.80	*D*_x_ = 1.657 Mg m^−^^3^
Monoclinic, *P*2_1_/*c*	Mo *K*α radiation, λ = 0.71073 Å
Hall symbol: -P 2ybc	Cell parameters from 1852 reflections
*a* = 18.7737 (14) Å	θ = 2.3–22.6°
*b* = 6.2837 (5) Å	µ = 2.51 mm^−^^1^
*c* = 15.7591 (12) Å	*T* = 291 K
β = 108.668 (1)°	Block, colourless
*V* = 1761.3 (2) Å^3^	0.30 × 0.26 × 0.24 mm
*Z* = 2	

### Data collection

**Table:**

Bruker SMART APEX CCD diffractometer	3446 independent reflections
Radiation source: sealed tube	2473 reflections with *I* > 2σ(*I*)
graphite	*R*_int_ = 0.044
φ and ω scans	θ_max_ = 26.0°, θ_min_ = 2.6°
Absorption correction: multi-scan (*SADABS*; Bruker, 2000)	*h* = −21→22
*T*_min_ = 0.48, *T*_max_ = 0.55	*k* = −7→7
9144 measured reflections	*l* = −18→19

### Refinement

**Table:**

Refinement on *F*^2^	Primary atom site location: structure-invariant direct methods
Least-squares matrix: full	Secondary atom site location: difference Fourier map
*R*[*F*^2^ > 2σ(*F*^2^)] = 0.057	Hydrogen site location: inferred from neighbouring sites
*wR*(*F*^2^) = 0.131	H-atom parameters constrained
*S* = 1.03	*w* = 1/[σ^2^(*F*_o_^2^) + (0.06*P*)^2^ + 1.22*P*] where *P* = (*F*_o_^2^ + 2*F*_c_^2^)/3
3446 reflections	(Δ/σ)_max_ < 0.001
223 parameters	Δρ_max_ = 0.29 e Å^−^^3^
0 restraints	Δρ_min_ = −0.46 e Å^−^^3^

### Special details

**Table:**

Geometry. All e.s.d.'s (except the e.s.d. in the dihedral angle between two l.s. planes) are estimated using the full covariance matrix. The cell e.s.d.'s are taken into account individually in the estimation of e.s.d.'s in distances, angles and torsion angles; correlations between e.s.d.'s in cell parameters are only used when they are defined by crystal symmetry. An approximate (isotropic) treatment of cell e.s.d.'s is used for estimating e.s.d.'s involving l.s. planes.
Refinement. Refinement of *F*^2^ against ALL reflections. The weighted *R*-factor *wR* and goodness of fit *S* are based on *F*^2^, conventional *R*-factors *R* are based on *F*, with *F* set to zero for negative *F*^2^. The threshold expression of *F*^2^ > σ(*F*^2^) is used only for calculating *R*-factors(gt) *etc*. and is not relevant to the choice of reflections for refinement. *R*-factors based on *F*^2^ are statistically about twice as large as those based on *F*, and *R*- factors based on ALL data will be even larger.

### Fractional atomic coordinates and isotropic or equivalent isotropic displacement parameters (Å^2^)

**Table:**

	*x*	*y*	*z*	*U*_iso_*/*U*_eq_	
Br1	0.22648 (3)	1.13983 (10)	0.17778 (4)	0.0651 (2)	
C1	0.3958 (3)	0.6851 (8)	0.1070 (3)	0.0477 (11)	
C2	0.3228 (3)	0.6150 (9)	0.1013 (4)	0.0604 (13)	
H2	0.3074	0.4775	0.0821	0.072*	
C3	0.2748 (2)	0.7509 (8)	0.1242 (3)	0.0481 (11)	
H3	0.2271	0.7041	0.1213	0.058*	
C4	0.2961 (2)	0.9538 (8)	0.1511 (3)	0.0448 (10)	
C5	0.3665 (2)	1.0246 (8)	0.1571 (3)	0.0450 (10)	
H5	0.3805	1.1626	0.1767	0.054*	
C6	0.4171 (2)	0.8944 (7)	0.1345 (3)	0.0397 (9)	
C7	0.4892 (2)	0.9756 (8)	0.1354 (3)	0.0482 (11)	
H7	0.5018	1.1160	0.1523	0.058*	
C8	0.6080 (2)	0.9353 (7)	0.1133 (3)	0.0387 (9)	
C9	0.6512 (3)	0.7946 (8)	0.0856 (4)	0.0563 (13)	
H9	0.6325	0.6601	0.0658	0.068*	
C10	0.7223 (3)	0.8507 (8)	0.0870 (3)	0.0513 (12)	
H10	0.7512	0.7546	0.0671	0.062*	
C11	0.7506 (2)	1.0454 (7)	0.1170 (3)	0.0403 (10)	
C12	0.7072 (2)	1.1915 (7)	0.1430 (3)	0.0457 (11)	
H12	0.7257	1.3271	0.1611	0.055*	
C13	0.6353 (2)	1.1350 (8)	0.1421 (3)	0.0476 (12)	
H13	0.6059	1.2315	0.1608	0.057*	
Mg1	0.0000	0.5000	0.0000	0.0372 (4)	
N1	0.53591 (18)	0.8598 (6)	0.1135 (2)	0.0436 (9)	
O1	0.44163 (19)	0.5502 (6)	0.0844 (3)	0.0727 (12)	
H1A	0.4853	0.6268	0.0825	0.109*	
O2	0.85670 (16)	1.0353 (5)	0.04402 (18)	0.0401 (7)	
O3	0.89136 (16)	0.9951 (6)	0.20297 (19)	0.0522 (8)	
O4	0.85143 (17)	1.3394 (5)	0.1372 (2)	0.0450 (7)	
O5	0.04046 (16)	0.9650 (5)	0.19393 (18)	0.0438 (7)	
H5D	−0.0040	0.9738	0.1965	0.053*	
H5A	0.0679	0.8973	0.2393	0.066*	
O11	0.04471 (18)	0.3651 (5)	0.12539 (18)	0.0486 (8)	
H11A	0.0911	0.2950	0.1291	0.073*	
H11C	0.0542	0.4746	0.1701	0.073*	
O12	0.05566 (19)	0.7759 (5)	0.0447 (2)	0.0503 (8)	
H12A	0.1036	0.7722	0.0349	0.075*	
H12B	0.0631	0.7904	0.1076	0.075*	
O13	−0.08843 (18)	0.6110 (5)	0.0402 (2)	0.0506 (8)	
H13A	−0.1230	0.6882	−0.0083	0.076*	
H13C	−0.1138	0.4923	0.0560	0.076*	
S1	0.84436 (5)	1.11031 (16)	0.12566 (6)	0.0322 (2)	

### Atomic displacement parameters (Å^2^)

**Table:**

	*U*^11^	*U*^22^	*U*^33^	*U*^12^	*U*^13^	*U*^23^
Br1	0.0464 (3)	0.0689 (4)	0.0887 (4)	0.0068 (2)	0.0338 (3)	0.0030 (3)
C1	0.040 (2)	0.047 (3)	0.055 (3)	−0.007 (2)	0.014 (2)	−0.003 (2)
C2	0.046 (3)	0.048 (3)	0.087 (4)	−0.018 (2)	0.021 (3)	−0.005 (3)
C3	0.028 (2)	0.058 (3)	0.056 (3)	−0.003 (2)	0.0105 (19)	0.013 (2)
C4	0.030 (2)	0.051 (3)	0.053 (3)	−0.0018 (19)	0.0128 (19)	0.004 (2)
C5	0.039 (2)	0.039 (3)	0.059 (3)	0.0022 (19)	0.019 (2)	0.002 (2)
C6	0.029 (2)	0.043 (3)	0.046 (2)	0.0004 (17)	0.0107 (17)	0.0030 (19)
C7	0.038 (2)	0.046 (3)	0.063 (3)	−0.003 (2)	0.018 (2)	−0.003 (2)
C8	0.0305 (19)	0.040 (2)	0.047 (2)	−0.0055 (17)	0.0137 (18)	−0.0009 (18)
C9	0.050 (3)	0.035 (2)	0.093 (4)	−0.020 (2)	0.035 (3)	−0.028 (2)
C10	0.048 (3)	0.049 (3)	0.062 (3)	−0.008 (2)	0.025 (2)	−0.025 (2)
C11	0.038 (2)	0.048 (3)	0.035 (2)	−0.0006 (19)	0.0116 (18)	0.0036 (18)
C12	0.041 (2)	0.039 (3)	0.067 (3)	−0.0133 (19)	0.031 (2)	−0.026 (2)
C13	0.047 (2)	0.049 (3)	0.063 (3)	−0.015 (2)	0.041 (2)	−0.031 (2)
Mg1	0.0380 (10)	0.0361 (11)	0.0405 (10)	0.0008 (8)	0.0166 (8)	−0.0003 (8)
N1	0.0279 (17)	0.048 (2)	0.054 (2)	−0.0080 (16)	0.0114 (15)	−0.0044 (18)
O1	0.0431 (18)	0.057 (2)	0.124 (3)	−0.0094 (17)	0.035 (2)	−0.026 (2)
O2	0.0475 (16)	0.0432 (17)	0.0386 (14)	−0.0031 (13)	0.0264 (13)	−0.0058 (13)
O3	0.0353 (16)	0.078 (2)	0.0378 (16)	0.0083 (16)	0.0042 (13)	0.0168 (16)
O4	0.0482 (17)	0.0379 (18)	0.0560 (18)	−0.0109 (14)	0.0267 (15)	−0.0102 (14)
O5	0.0436 (16)	0.0509 (19)	0.0358 (15)	0.0081 (14)	0.0111 (13)	0.0089 (13)
O11	0.063 (2)	0.0476 (19)	0.0329 (15)	0.0076 (15)	0.0119 (14)	0.0015 (13)
O12	0.065 (2)	0.0463 (19)	0.0510 (17)	−0.0202 (16)	0.0340 (16)	−0.0143 (14)
O13	0.0584 (19)	0.0361 (18)	0.072 (2)	0.0118 (15)	0.0415 (17)	0.0130 (15)
S1	0.0313 (5)	0.0350 (6)	0.0320 (5)	−0.0036 (4)	0.0123 (4)	0.0004 (4)

### Geometric parameters (Å, °)

**Table:**

Br1—C4	1.899 (5)	C11—S1	1.770 (4)
C1—O1	1.334 (6)	C12—C13	1.390 (6)
C1—C6	1.402 (6)	C12—H12	0.9300
C1—C2	1.415 (6)	C13—H13	0.9300
C2—C3	1.370 (7)	Mg1—O12^i^	2.031 (3)
C2—H2	0.9300	Mg1—O12	2.031 (3)
C3—C4	1.362 (7)	Mg1—O11	2.065 (3)
C3—H3	0.9300	Mg1—O11^i^	2.065 (3)
C4—C5	1.367 (6)	Mg1—O13^i^	2.077 (3)
C5—C6	1.385 (6)	Mg1—O13	2.077 (3)
C5—H5	0.9300	O1—H1A	0.9600
C6—C7	1.443 (6)	O2—S1	1.457 (3)
C7—N1	1.268 (6)	O3—S1	1.449 (3)
C7—H7	0.9300	O4—S1	1.452 (3)
C8—C9	1.362 (6)	O5—H5D	0.8501
C8—C13	1.376 (6)	O5—H5A	0.8500
C8—N1	1.436 (5)	O11—H11A	0.9600
C9—C10	1.373 (6)	O11—H11C	0.9600
C9—H9	0.9300	O12—H12A	0.9601
C10—C11	1.358 (6)	O12—H12B	0.9599
C10—H10	0.9300	O13—H13A	0.9599
C11—C12	1.374 (6)	O13—H13C	0.9600
			
O1—C1—C6	122.3 (4)	C8—C13—C12	119.6 (4)
O1—C1—C2	118.6 (4)	C8—C13—H13	120.2
C6—C1—C2	119.1 (4)	C12—C13—H13	120.2
C3—C2—C1	119.6 (5)	O12^i^—Mg1—O12	180.0
C3—C2—H2	120.2	O12^i^—Mg1—O11	89.32 (13)
C1—C2—H2	120.2	O12—Mg1—O11	90.68 (13)
C4—C3—C2	120.9 (4)	O12^i^—Mg1—O11^i^	90.68 (13)
C4—C3—H3	119.6	O12—Mg1—O11^i^	89.32 (13)
C2—C3—H3	119.6	O11—Mg1—O11^i^	180.0
C3—C4—C5	120.5 (4)	O12^i^—Mg1—O13^i^	88.86 (13)
C3—C4—Br1	119.4 (3)	O12—Mg1—O13^i^	91.14 (13)
C5—C4—Br1	120.1 (4)	O11—Mg1—O13^i^	91.94 (13)
C4—C5—C6	121.1 (4)	O11^i^—Mg1—O13^i^	88.06 (13)
C4—C5—H5	119.4	O12^i^—Mg1—O13	91.14 (13)
C6—C5—H5	119.4	O12—Mg1—O13	88.86 (13)
C5—C6—C1	118.8 (4)	O11—Mg1—O13	88.06 (13)
C5—C6—C7	120.5 (4)	O11^i^—Mg1—O13	91.94 (13)
C1—C6—C7	120.6 (4)	O13^i^—Mg1—O13	180.0
N1—C7—C6	121.5 (4)	C7—N1—C8	123.0 (4)
N1—C7—H7	119.3	C1—O1—H1A	108.7
C6—C7—H7	119.3	H5D—O5—H5A	109.5
C9—C8—C13	120.0 (4)	Mg1—O11—H11A	109.3
C9—C8—N1	116.4 (4)	Mg1—O11—H11C	109.3
C13—C8—N1	123.6 (4)	H11A—O11—H11C	109.5
C8—C9—C10	120.2 (4)	Mg1—O12—H12A	109.1
C8—C9—H9	119.9	Mg1—O12—H12B	109.2
C10—C9—H9	119.9	H12A—O12—H12B	109.5
C11—C10—C9	120.4 (4)	Mg1—O13—H13A	109.3
C11—C10—H10	119.8	Mg1—O13—H13C	109.2
C9—C10—H10	119.8	H13A—O13—H13C	109.5
C10—C11—C12	120.2 (4)	O3—S1—O4	112.5 (2)
C10—C11—S1	120.2 (4)	O3—S1—O2	110.78 (19)
C12—C11—S1	119.6 (3)	O4—S1—O2	113.44 (18)
C11—C12—C13	119.5 (4)	O3—S1—C11	105.83 (19)
C11—C12—H12	120.3	O4—S1—C11	106.7 (2)
C13—C12—H12	120.3	O2—S1—C11	107.05 (19)

Symmetry codes: (i) −*x*, −*y*+1, −*z*.

### Hydrogen-bond geometry (Å, °)

**Table:**

*D*—H···*A*	*D*—H	H···*A*	*D*···*A*	*D*—H···*A*
O1—H1A···N1	0.96	1.73	2.570 (5)	144
O5—H5D···O3^ii^	0.85	2.00	2.854 (4)	180
O5—H5A···O4^iii^	0.85	2.08	2.892 (4)	160
O11—H11A···Br1^iv^	0.96	2.60	3.539 (3)	166
O11—H11C···O3^iii^	0.96	1.94	2.710 (4)	136
O12—H12A···O2^v^	0.96	2.04	2.747 (4)	129
O12—H12B···O5	0.96	1.90	2.729 (4)	143
O13—H13C···O4^vi^	0.96	1.88	2.763 (4)	152

Symmetry codes: (ii) *x*−1, *y*, *z*; (iii) −*x*+1, *y*−1/2, −*z*+1/2; (iv) *x*, *y*−1, *z*; (v) −*x*+1, −*y*+2, −*z*; (vi) *x*−1, *y*−1, *z*.
